# Toxic Haemorrhagic Colitis: A Rare Presentation of Eosinophilic Colitis

**DOI:** 10.1155/2012/279813

**Published:** 2012-09-10

**Authors:** Shen-Ann Eugene Yeo, Yon Kuei Lim, Kiat Hon Tony Lim, Choong-Leong Tang

**Affiliations:** ^1^Department of Colorectal Surgery, Singapore General Hospital, 169608, Singapore; ^2^Department of Pathology, Singapore General Hospital, 169608, Singapore

## Abstract

Eosinophilic colitis is a rare condition that usually presents with non specific abdominal symptoms. Very uncommonly it presents with an acute surgical emergency such as peritonitis or haemorrhage. We present a rare presentation of eosinophilic colitis with toxic hemorrhagic colitis and ischaemic bowel requiring laparotomy and bowel resection.

## 1. Introduction

Eosinophilic colitis (EC) is a rare clinical entity [1]. Its causes may be divided into primary and secondary, with a broad list of differential diagnoses. The clinical presentation is usually nonspecific. Severe cases may develop severe abdominal pain and distension, malabsorption, intestinal obstruction, and even gastrointestinal haemorrhage [2]. Once diagnosed, EC responds to steroid therapy. However, due to the nonspecific nature of the symptoms and the rarity of the condition, it is commonly diagnosed only late in presentation.

We discuss a rare case of severe eosinophilic colitis presenting as haemorrhagic colitis requiring laparotomy and bowel resection.

## 2. Case Presentation

A 45-year-old Chinese male presented to the hospital with complaints of a 4-day history of fever, abdominal pain, nausea, and vomiting, as well as mild bloody diarrhea.

On examination, he was febrile but otherwise stable. Abdominal examination revealed generalized mild tenderness, and per-rectal examination revealed slight blood stains and watery stool. His white blood cell count was raised at 16.1 × 10(9)/L. He was treated as for severe gastroenteritis with dehydration with intravenous fluids and antibiotics and subsequently improved clinically. Blood and stool cultures were negative.

**Figure 1:**
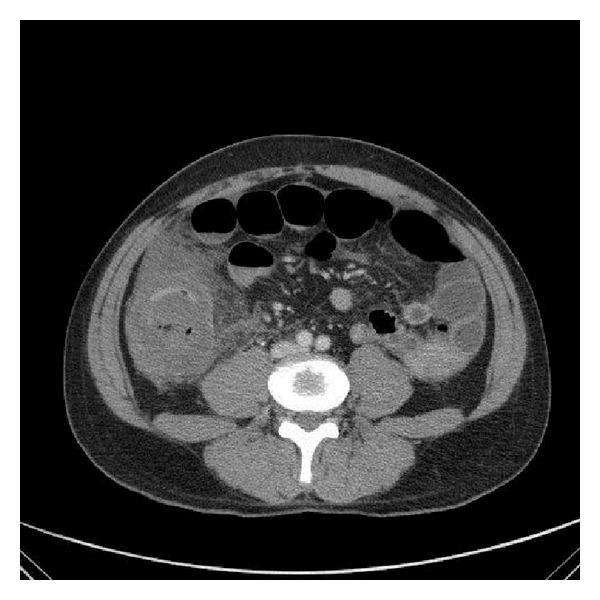
Ascending colon inflammation.

**Figure 2:**
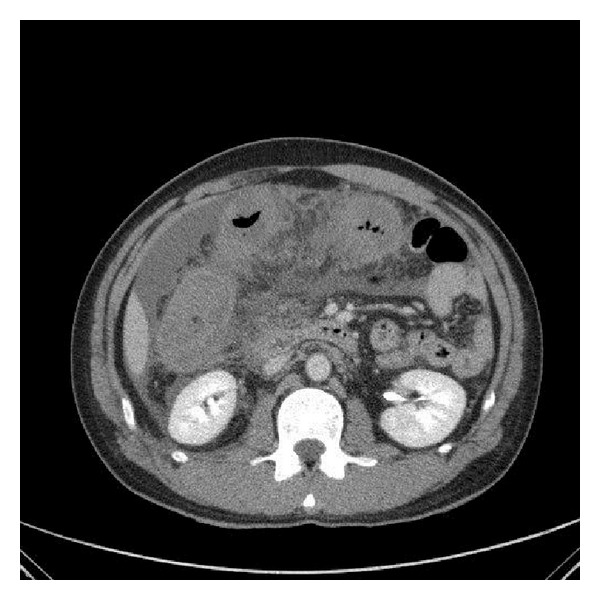
Transverse colon inflammation.

**Figure 3:**
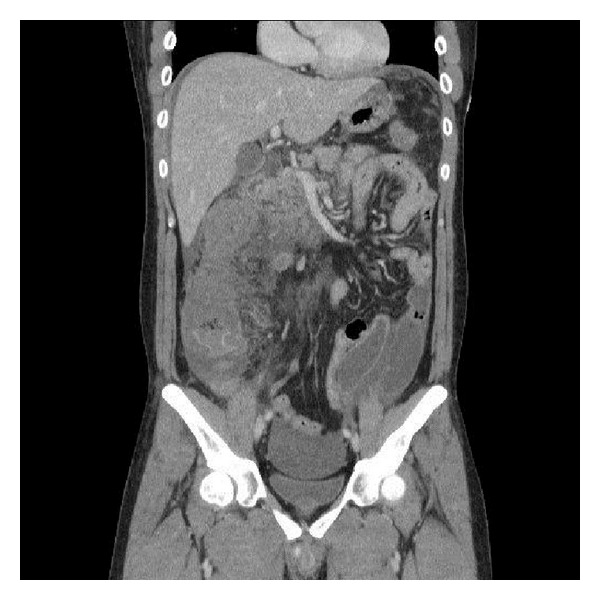
Coronal view and pelvic free fluid.

On the 3rd day of admission, he developed increasing abdominal pain and distension, with a rise in the white cell counts to 17.1 × 10(9)/L. His eosinophil counts also rose to 17.0%. An urgent computed tomogram (CT) of the abdomen and pelvis was ordered which showed a large amount of ascites, as well as a long segment of circumferential mural thickening and poor enhancement of the ascending colon and proximal transverse colon suggesting ischaemic bowel (Figures 1, 2, and 3).

An emergency laparotomy was performed. Intraoperatively, there was 2 litres of hemoperitoneum from severe haemorrhagic colitis and transmural infarction involving the right and proximal transverse colon. An extended right hemicolectomy and double-barreled stoma were performed.

**Figure 4:**
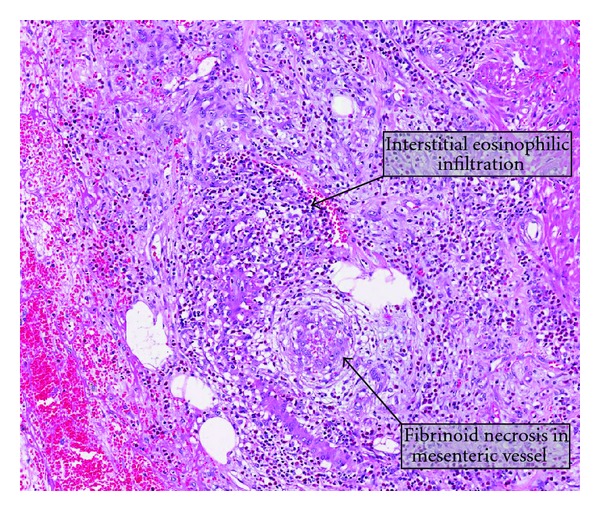
Eosinophilic colitis histology.

Histology revealed ischaemic bowel with eosinophilic inflammation and vasculitis involving the mesenteric vessels (Figure 4), with patchy transmural haemorrhagic infarction and eosinophilic microabscesses present. Nine lymph nodes showed eosinophilic infiltration. The overall picture is consistent with that of eosinophilic vasculitis and colitis.

## 3. Discussion

Haemorrhagic colitis has many different etiologies, including infectious colitis with either bacterial or parasitic infections. Other causes include ischaemic colitis, as well as inflammatory bowel disease such as Crohn's colitis and ulcerative colitis. Eosinophilic colitis (EC) is a rare cause of colitis; however, it may very occasionally present with haemorrhagic manifestations.

Primary eosinophilic gastrointestinal disease (EGID) was first described by Kaiser in 1937 [3]. It is a rare chronic inflammatory bowel condition of unknown etiology, characterized by inflammatory changes rich in eosinophils without evidence of other known causes of eosinophilia. This may affect any segment of the gastrointestinal tract and presents in a myriad of different manifestations.

Causes of EC may be divided into 2 main groups, that is, those with primary and secondary causes. Primary eosinophilic colitis is the rarest manifestation of EGID, the other more common presentations being eosinophilic esophagitis and eosinophilic gastroenteritis [4]. The disease mainly affects the paediatric population, although it has been reported in the adult population as well. The hallmarks of EC are peripheral eosinophilia, segmental eosinophilic colonic infiltration, and functional abnormalities [5]. Secondary causes of EC include parasitic infection, inflammatory bowel disease, carcinoma, connective tissue disease, drug-related causes such as Rifampicin, Clozapine, and Carbamazepine, and vasculitic causes.

Clinical presentation of the primary EC depends on the layer of intestinal wall that is infiltrated by eosinophils and correlates well with the physical symptoms and clinical findings. Mucosal predominant disease presents with diarrhoea, protein wasting, and malabsorption [6]. Transmural disease is associated with thickened bowel, obstruction, perforation, volvulus, intussusception [7], and perforation [8]. Serosal involvement is characterized by presence of eosinophilic ascites [9]. However, there has been no report so far in the literature on eosinophilic colitis presenting with haemorrhagic colitis.

The diagnosis of EC is made via the presence of symptoms, peripheral eosinophilia, endoscopic or histological findings, and eosinophilic ascitis, with exclusion of all other causes of secondary eosinophilia. Histological findings show eosinophilic infiltrates throughout the lamina propria, with extension through the muscularis mucosa into the submucosa. Crypt abscesses and lymphonodular hyperplasia may also be evident [10].

Treatment of EC has not been well established due to the low numbers of reported cases worldwide. In neonates, there has been some beneficial effect of withdrawing specific food triggers for cases with food allergies; however, in adults, the response of dietary modification is less clearer. In patients who present with abdominal symptoms, firstly indications for surgical exploration must be ruled out, such as free intraabdominal air, volvulus, obstruction, or peritonitis. Patients who present as such should be urgently brought to the operating theater for a laparotomy and assessment of the bowel intraoperatively, and resection with or without primary anastomosis depending on the intraoperative findings. In all other patients with no such indications, corticosteroids have been found to be effective for symptom control in EC [11, 12]. The majority of cases respond within 2 weeks of treatment; however, relapse is frequent and may require recurrent courses which may lead to steroid dependence. Budesonide has been shown to be effective in cases of EC, particularly in the right colon [13]. However, it must be emphasized again that surgical indications, as well as parasitic infection and drug causes, are essential to be ruled out before starting corticosteroids, as in these cases, treatment will certainly worsen the patient's condition.

In the above-mentioned case, the patient was extensively worked up and treated empirically for various infective causes of haemorrhagic colitis. Unfortunately, he rapidly deteriorated and developed ischaemic haemorrhagic bowel which necessitated emergency surgery, only after which the diagnosis of eosinophilic colitis was made on histological examination of the resected specimen.

## 4. Conclusion

Eosinophilic colitis is a rare condition. It may present acutely and give rise to potentially severe and fatal complications like haemorrhage and perforation. With early diagnosis and appropriate treatment, we may be able to avoid serious complications in such patients.
